# Cytokine absorption during human kidney perfusion reduces delayed graft function–associated inflammatory gene signature

**DOI:** 10.1111/ajt.16371

**Published:** 2020-11-22

**Authors:** John R. Ferdinand, Sarah A. Hosgood, Tom Moore, Ashley Ferro, Christopher J. Ward, Tomas Castro‐Dopico, Michael L. Nicholson, Menna R. Clatworthy

**Affiliations:** ^1^ Molecular Immunity Unit University of Cambridge Department of Medicine Laboratory of Molecular Biology Cambridge UK; ^2^ National Institute of Health Research Blood and Transplant Research Unit in Organ Donation Cambridge UK; ^3^ University of Cambridge Department of Surgery Cambridge UK

**Keywords:** clinical research / practice, delayed graft function (DGF), donors and donation: deceased, kidney (allograft) function / dysfunction, kidney disease: immune / inflammatory, kidney transplantation / nephrology, organ perfusion and preservation, translational research / science

## Abstract

Transplantation is the optimal treatment for most patients with end‐stage kidney disease but organ shortage is a major challenge. Normothermic machine perfusion (NMP) has been used to recondition marginal organs; however, mechanisms by which NMP might benefit organs are not well understood. Using pairs of human kidneys obtained from the same donor, we compared the effect of NMP with that of cold storage on the global kidney transcriptome. We found that cold storage led to a global reduction in gene expression, including inflammatory pathway genes and those required for energy generation processes, such as oxidative phosphorylation (OXPHOS). In contrast, during NMP, there was marked upregulation OXPHOS genes, but also of a number of immune and inflammatory pathway genes. Using biopsies from kidneys undergoing NMP that were subsequently transplanted, we found that higher inflammatory gene expression occurred in organs with prolonged delayed graft function (DGF). Therefore, we used a hemoadsorber (HA) to remove pro‐inflammatory cytokines. This attenuated inflammatory gene expression increased OXPHOS pathway genes and had potentially clinically important effects in reducing the expression of a DGF‐associated gene signature. Together, our data suggest that adsorption of pro‐inflammatory mediators from the perfusate represents a potential intervention which may improve organ viability.

## Abstract 2

A gene signature associated with reduced delayed graft function after kidney transplantation that increases during machine normothermic perfusion further increases if cytokines are removed from the perfusate during perfusion.

**Abbreviations**
DBD: Deceased brain death
DCD: Deceased circulatory death
DGF: Delayed graft function
ECD: Extended criteria donors
HSP: Heat Shock proteins
HA: Hemoadsorber
IL: Interleukin
NMP: Normothermic machine perfusion
OXPHOS: Oxidative phosphorylation

## 1 INTRODUCTION

Kidney transplantation represents the optimal treatment for most patients with end‐stage kidney disease, with benefits for both quality and quantity of life.^1^ Organ shortage is a major challenge, and several strategies have been employed to increase the number of kidneys available, including the use of deceased circulatory death (DCD) donors and extended criteria donors (ECD), both of which are associated with higher rates of delayed graft function (DGF) compared with deceased brainstem death (DBD) donor.^2^, ^3^ In DCD kidneys, exposure to warm ischemia during the process of circulatory cessation makes a significant contribution to DGF. DGF occurs due to ischemic tubular cell damage or death, which can stimulate innate immune activation via NLRP3 inflammasome assembly leading to the generation of interleukin (IL)1β and IL18.^4^, ^5^, ^6^ Indeed, the presence of inflammatory cytokines in urine has been used as a biomarker of acute kidney injury and DGF.^7^, ^8^, ^9^

Normothermic machine perfusion (NMP) allows transplanted organs to be perfused with warm, oxygenated red blood cells, in the absence of circulating immune components, including complement and neutrophils.^10^, ^11^, ^12^, ^13^, ^14^ This process has been used to assess marginal organs^15^ and to “recondition” organs to facilitate the transplantation of kidneys that were initially declined following offer via national organ allocation service.^16^ Our previous experience using NMP provided the rationale for a randomized controlled trial to assess its efficacy in preventing DGF in DCD kidneys,^17^ but the mechanisms by which NMP might benefit transplant kidneys are not fully understood. Furthermore, the question of whether additional manipulation of the kidney during NMP, for example by removal of pro‐inflammatory cytokines and chemokines from the perfusate, might offer additional benefits in optimizing the organ prior to transplantation has not been addressed in human kidneys, but our study of porcine NMP demonstrated promising results.^18^

Here we took an unbiased approach to address these two questions using transcriptomic analysis of human kidney biopsies taken at the start and end of NMP to assess global changes in gene expression. We used pairs of human kidneys from a single donor enabling a comparison of the effect of different interventions in organs with an identical genetic background and were able to assess the impact of those changes on a prediction of graft outcome.

## 2 RESULTS

### 2.1 Paired kidneys are genetically similar and a useful model for assessing interventions

We investigated the transcriptional changes associated with organ preservation and NMP in two independent studies, each with five pairs of kidneys, making a total of 10 pairs/20 human kidneys. Of these, two were from DBD and eight from DCD donors (Table S1, S2 and Figure [Link], [Link], [Link], [Link]). In these studies, we took cortical biopsies pre‐ and postintervention (cold storage or NMP) and investigated the transcriptional landscape using RNA sequencing (RNA‐Seq). The use of paired kidneys and paired biopsies from the same kidney allowed us to control for biological variation and to regress this confounder out of the analysis (Figure [Link], [Link], [Link], [Link]). We confirmed that kidney pairs start with a common transcriptional landscape (Fig. S2B) and by applying different interventions to each kidney in a pair, we are able to study the effect of an intervention independent of biological variation.

### 2.2 NMP results in an increase in OXPHOS and inflammatory pathway genes compared with cold storage

**Figure 1:**
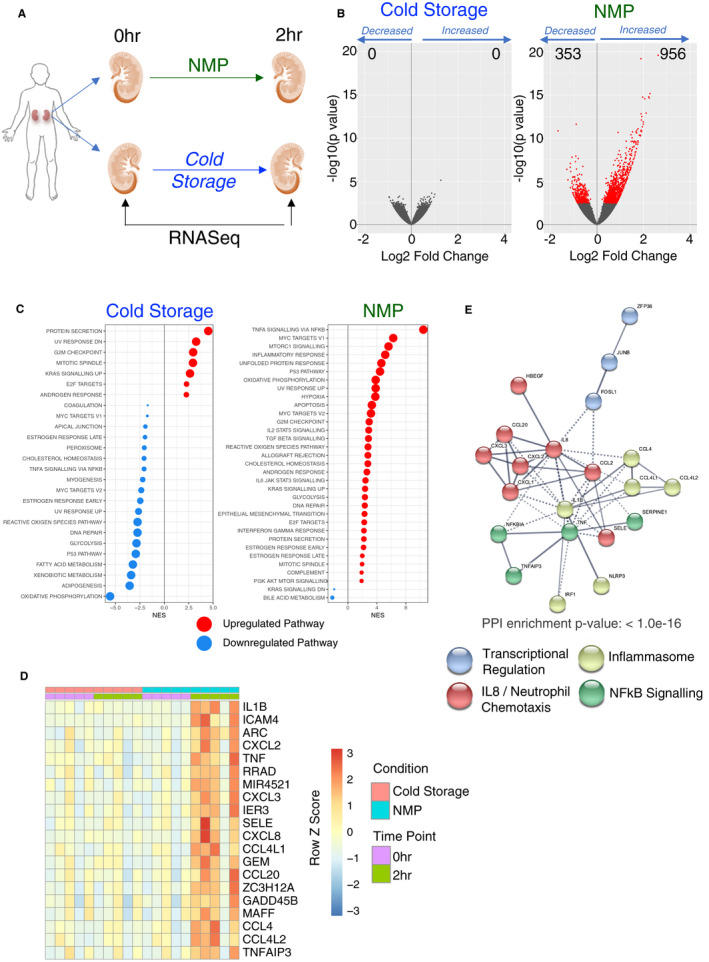
Kidneys exposed to cold storage show limited changes in gene expression compared with those undergoing NMP. A, Pairs of kidneys were obtained which had been declined for use in transplantation. One kidney was maintained in static cold storage and the other underwent normothermic machine perfusion (NMP). Biopsies were taken from the outer cortex at the start and after 2 hours. B, Volcano plot indicating change in gene expression at 2 hours for the indicated group compared to the start. Red dots indicate differentially expressed genes with an adjusted *P* value <0.05 and the experimental group is indicated above the plot. C, Gene set enrichment analyses of the differential expressions from B against the hallmarks pathways. Only significant pathways (FDR q value <0.05) are plotted. Red dots indicate positive enrichment and blue negative, the size of the dot is inversely correlated with the FDR q value and the position indicates the normalized enrichment score (NES). D, Heatmap of the top 20 significantly upregulated genes during NMP, genes are ranked by log2 fold change. E, STRING analysis of the top 50 genes upregulated during NMP. The color of each node indicates membership of each cluster

To investigate the potential mechanisms by which NMP might impact kidneys, we initially took five kidney pairs (n = 4 DCD donors and n = 1 deceased brainstem death [DBD] donor, Figure [Link], [Link], [Link], [Link], Table [Link], [Link], [Link], [Link]) and performed a time 0 (0 hour) cortical biopsy. At this point, the kidneys were randomized to static cold storage or NMP, as described previously ^10^ (Figure 1A). After 2 hours, a second biopsy was taken from both kidneys and RNA‐Seq performed. When comparing gene expression between the time 0‐ and 2‐hour biopsies, we found that kidneys placed in static cold storage had no statistically significant change in the expression of any individual gene when corrected for multiple testing (Figure 1B, left panel). In contrast, during the course of 2 hours of NMP, 956 genes were upregulated and 353 genes were downregulated (Figure 1B, right panel). We next assessed changes in the expression of groups of genes within a common pathway, comparing tens or hundreds of genes rather than any individual gene, thereby further reducing the effect of biological interindividual variation on the analysis. This revealed changes in functionally important pathways, where the expression of each individual gene within that pathway was not altered with sufficient magnitude to reach statistical significance. This gene set enrichment analysis (GSEA) demonstrated that cold storage had a substantial impact on a number of metabolic pathways (Figure 1C, left panel). In particular, there was a marked reduction in genes involved in oxidative phosphorylation (OXPHOS), a key pathway required to generate ATP.^19^ In contrast, OXPHOS was among the pathways significantly upregulated during NMP (Figure 1C, right panel), with potential benefits for cell viability and the restoration of cellular homeostasis. In addition, a number of pathways involved in immune or inflammatory processes were induced during NMP, with “TNFα signaling via NFkB” demonstrating the largest increase. In keeping with this, *TNF* as well as *IL1B* and the neutrophil‐recruiting chemokines *CXCL8* (IL8) and *CXCL2* were among the most upregulated genes in 2‐hour NMP biopsies (Figure 1D). String analysis of the top 50 upregulated genes revealed upregulation of biochemically related genes which were clustered into four major nodes; IL8 and neutrophil‐recruiting chemokines, Inflammasome‐associated genes, NFkB signaling, and transcriptional regulation (Figure 1E). Of note, DBD and DCD kidneys were transcriptionally similar at baseline (Figure [Link], [Link], [Link], [Link]), and the gene pathways changing during NMP were similar in DBD and DCD kidneys (Figure [Link], [Link], [Link], [Link]). Together, our analysis demonstrates that during NMP, there is an increased expression of genes that promote the generation of energy, with potentially beneficial effects for the organ, but a simultaneous induction of pro‐inflammatory genes, which may be deleterious.

### 2.3 Inflammatory pathway genes in NMP kidneys associated with prolonged delayed graft function

**Figure 2:**
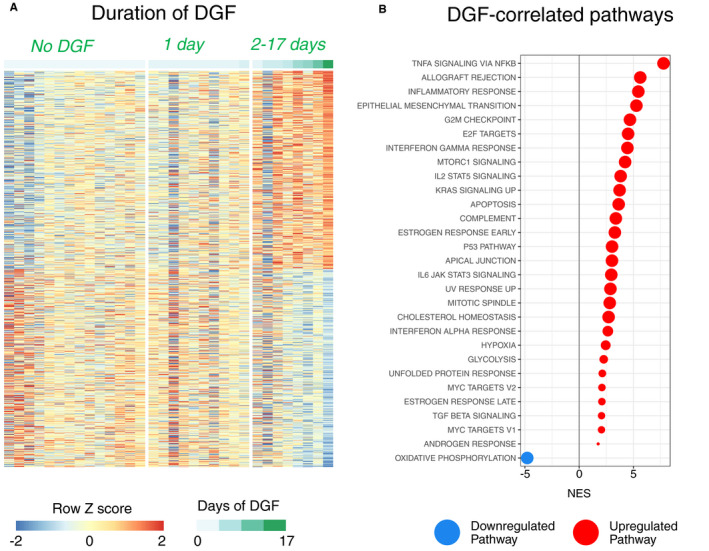
Correlation of transcriptome following NMP with the length of delayed graft function. A, Gene expression was correlated with the length of DGF in biopsies taken following NMP as part of a randomized clinical trial. The expression levels of the 1000 genes with the greatest correlation with outcome have been plotted. B, GSEA was performed for the correlation from A against the hallmarks data set. Only significant pathways (FDR q value <0.05) are plotted. Red dots indicate positive enrichment and blue negative, the size of the dot is inversely correlated with the FDR q value and the position indicates the normalized enrichment score (NES)

In order to link the transcriptional changes occurring during cold storage and NMP to clinical outcomes, we performed RNA‐Seq on biopsies taken from 33 DCD kidneys that had undergone NMP as part of a randomized clinical trial currently assessing its efficacy.^17^ Samples were available on a subset of kidneys randomized to the NMP arm of the study that were subsequently transplanted (Table [Link], [Link], [Link], [Link], Figure [Link], [Link], [Link], [Link]). DGF is more common in DCD kidneys and is classically defined as a requirement for dialysis in the first week posttransplant. However, in the immediate posttransplant period, some patients receive a single episode of dialysis for hyperkalemia, which does not necessarily reflect the presence of significant acute tubular necrosis. We therefore used a model correlating gene expression with time between transplant surgery and the last dialysis session (ie, duration of DGF). This showed more marked transcriptional changes in transplants requiring dialysis beyond the first 24 hours of posttransplant (Figure 2A), as well as a significant positive correlation between the expression of inflammatory pathway genes including “TNFA signaling via NFkB” and “inflammatory response” pathways and the length of DGF, with a greater enrichment of these pathways in kidneys that experienced more prolonged DGF posttransplant (Figure 2B). Conversely, the length of DGF negatively correlated with the magnitude of expression of “OXPHOS” pathway genes (Figure 2B). Taken, together these data indicate that following NMP, kidneys that have lower expression of inflammatory pathway genes and higher expression of “OXPHOS” pathway genes are less susceptible to prolonged DGF posttransplant. This would support the conclusion that the molecular changes occurring during NMP may have both beneficial (induction of OXPHOS pathway genes) and deleterious (induction of inflammatory pathways genes) effects.

### 2.4 Urine output and renal blood flow during NMP demonstrate differing associations with OXPHOS and inflammatory pathway genes

**Figure 3:**
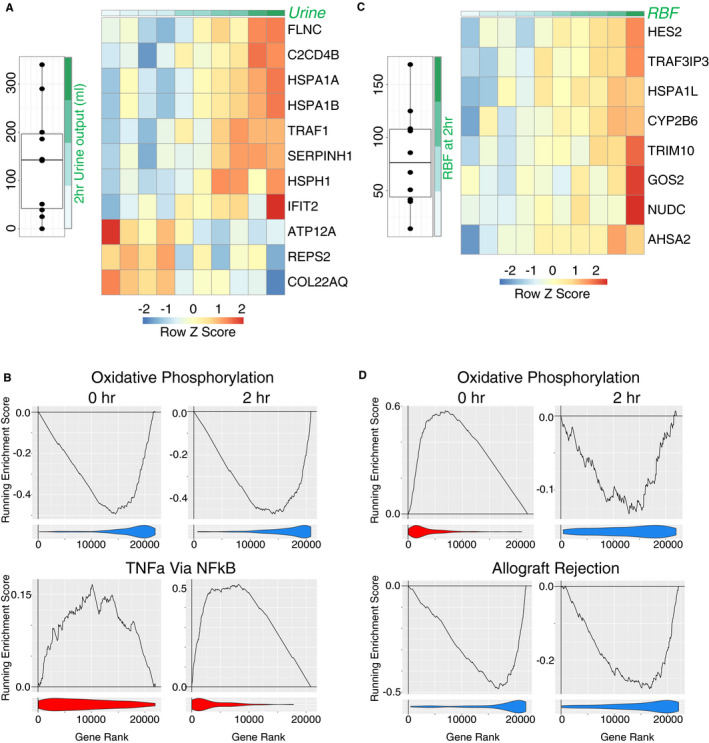
Correlation of transcriptome of NMP after 2 hours with perfusion parameters. A, left panel, Total urine output by each kidney after 2 hours of normothermic machine perfusion (NMP). Right panel, Heatmap of all genes that are significantly correlated with urine output in biopsies taken post‐NMP. B, Enrichment plots from GSEA for key pathways from the Hallmark database. Analysis is for the correlation of urine output after 2 hours with the transcriptome of the kidney either pre–(0‐hr) or post–2‐hr NMP. The line indicates the running enrichment score and the violin plot indicates the distribution of the member genes of the geneset throughout the ranked gene list used in each analysis. C, left panel, Renal blood flow after 2 hours of NMP. Right panel, Heatmap of all genes which significantly correlate with 2‐hr RBF in samples taken after 2‐hr NMP. D, Enrichment plots for correlation of transcriptome with renal blood flow as for B

The quantity of urine produced during NMP is one of a number of parameters included in surgical quality assessment scores used to guide organ utilization decisions,^15^ but whether high urine output during NMP truly portends a good prognosis for the kidney and the underlying molecular pathways activated in kidneys with a high urine output is unclear. In a total of 10 kidneys undergoing NMP (NMP only kidneys, Table S1 and S2, Figure [Link], [Link], [Link], [Link]), we observed a range of urine outputs from 0 to 340 ml over the 2‐hour period of perfusion (Figure 3A left panel). Of note, when we compared the pathways changing during NMP in five kidneys in two independent experiments, we found that similar pathways were induced (Figure [Link], [Link], [Link], [Link]) confirming the reproducibility of our experimental design and the utility of comparing pathways rather than individual genes. In the 2‐hour postperfusion biopsies, the expression of 11 genes significantly correlated with the urine volume produced during this period. These included heat shock proteins (HSPs), HSPA1A, HSPA1B, and HSPH1, which positively correlated with increased urine output (Figure 3A right panel). Gene set enrichment analysis showed that urine output also positively correlated with the “TNFα signaling via NFkB” pathway genes, and negatively correlated with OXPHOS genes in postperfusion biopsies (Figure 3B, S3C). Similarly, in preperfusion biopsies, OXPHOS negatively correlated with urine output in these kidneys while pathways associated with immune activation including “TNFα signaling via NFkB” and “allograft rejection” positively correlated with urine output, suggesting that NMP had little effect on these processes, or on other pathways analyzed, in high urine output kidneys (Figure 3B, S3C‐D). Since the induction of inflammatory pathway genes was observed in our clinical samples with prolonged DGF (Figure 2B), these data challenge the dogma that high urine output occurs in more viable, “healthy” kidneys and suggest that in fact these kidneys may have more inflammatory potential, are less able to generate energy, and that this is not substantially altered during NMP.

Renal blood flow during perfusion has also been assessed as a parameter that may reflect subsequent transplant function.^15^ In the 10 kidneys studied, renal blood flow at 2 hours of perfusion varied from 14.1 to 168.8 ml/minute (Figure 3C left panel). In the 2‐hour postperfusion biopsies, eight genes significantly positively correlated with increasing renal blood flow (Figure 3C right panel), including HSPA1L, but there was no direct overlap between this gene list and those correlating with urine output (Figure 3A). Gene set enrichment analysis demonstrated that high renal blood flow positively correlated with OXPHOS genes in 0‐hour biopsies, but in contrast to those with the highest urine output, perfusion had a significant impact, resulting in a negative correlation with “OXPHOS” pathway genes by 2 hours (Figure 3D, Figure [Link], [Link], [Link], [Link]). Renal blood flow negatively correlated with a number of immune and inflammatory gene pathways in both 0‐ and 2‐hour biopsies (Figure 3D, Figure S4B), in contrast to urine output (Figure 3B, Figure S3C). Together these data suggest that renal blood flow and urine output may not be equivalent indicators of a more viable kidney, but that the former may more faithfully identify a kidney less likely to have prolonged DGF.

### 2.5 Addition of a hemoadsorber to the perfusion circuit has no effect on perfusion parameters but significantly reduces inflammatory gene expression, including *NLRP3* and *IL1B*

**Figure 4:**
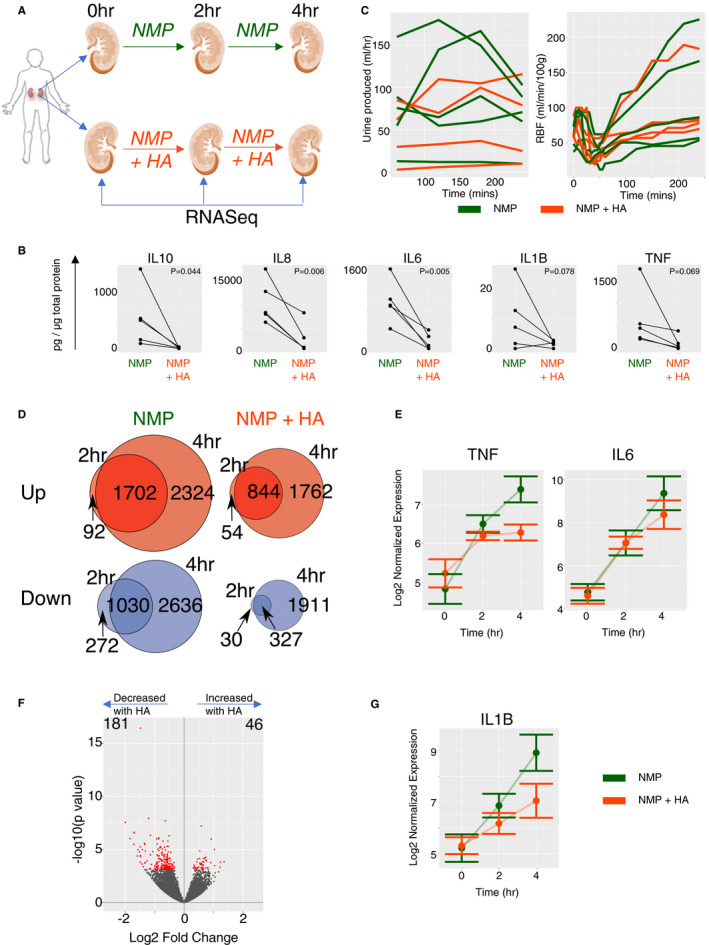
Addition of a hemoadsorber to the normothermic machine perfusion circuit has substantial effects on cytokine level in perfusate and the transcriptome but not on physical parameters recorded on the rig. A, Pairs of human kidneys were taken from the same donor, one of the pair underwent the standard normothermic machine perfusion (NMP) protocol for 4 hours and the other kidney underwent NMP with the addition of a hemoadsorber to the circuit. Samples were taken for RNA‐Seq prior to perfusion (0 hour), after 2 hours, and at the end (4 hours). B, Concentration of key cytokines in the perfusate after 4 hours of NMP. Line indicates pairs of kidneys. Cytokine measurements were normalized to total perfusate protein content. *P* value stated is from a paired *t* test testing for the reduction of cytokines with addition of HA. C, Urine production and renal blood flow (RBF) across the time course of perfusion. Green line indicates NMP alone and orange with the addition of hemoadsorber. D, Venn diagram showing the numbers of genes significantly differently expressed when comparing the 2‐hr or the 4‐hr samples to the pre(0‐hr) samples. Red diagrams are upregulated genes and blue downregulated. The intersection are the genes which are deferentially expressed at both time points in the same direction. E, log2 normalized expression values for the indicated genes across perfusion from the tissue transcriptome, standard error bars are indicated. F, Volcano plot for pairwise comparison of NMP alone with NMP +HA at 4 hours. Red indicate differentially expressed genes with adjusted *P* value <0.05. G, As for E

Analyses of kidney perfusate have demonstrated a substantial increase in the concentration of pro‐inflammatory cytokines and chemokines during the course of hypothermic and NMP.^20^, ^21^ These bioactive molecules recirculate into the kidney, with the potential to induce further inflammation. Given that our analysis of transplanted kidney samples indicated that the induction of TNF‐dependent genes in NMP kidneys was associated with DGF (Figure 2B), we hypothesized that removal of cytokines and chemokines from the perfusion circuit may attenuate inflammatory gene induction, with potential beneficial effects for the kidney. Such an approach has shown some efficacy in patients with systemic inflammatory response syndrome,^22^, ^23^ and was associated with increased renal blood flow in porcine kidneys undergoing NMP.^18^ To test this in human kidneys, we took an additional five kidney pairs and performed NMP for 4 hours with biopsies taken at 0, 2, and 4 hours (Figure [Link], [Link], [Link], [Link]). In each case, a cytosorb hemoadsorber (HA) that removes molecules with a molecular weight of 10‐50 kDa was added to the perfusion circuit of one kidney within each pair (NMP+HA) (Figure 4A). As anticipated, the addition of the HA resulted in lower concentrations of a variety of cytokines in the perfusate (Figure 4B) but had no effect on renal blood flow, urine output or composition, oxygen consumption, and acid–base homeostasis (Figure 4C, Table S4, S5). Thus, over 4 hours of NMP, the HA had no impact on the perfusion parameters currently used clinically to generate quality assessment scores but had a substantial effect on gene expression; Following NMP, 1794 and 4026 genes were upregulated at 2 and 4 hours, respectively, including *TNF* and *IL6* (Figure 4D,E). but only half this number (n =* *898 and n = 2606) were increased when the HA was present (Figure 4D, Figure S5A and B). The number of genes downregulated was also reduced by the addition of HA (Figure 4D). After 4 hours of NMP, 46 genes were significantly upregulated and 181 downregulated with the addition of the HA (Figure 4F). This attenuated transcriptional response included NLRP3 inflammasome activation‐associated genes, such as *IL1B*, *NLRP3*, and *CASP1* (Figure 4G, Figure [Link], [Link], [Link], [Link]) and some neutrophil‐recruiting chemokines (Figure [Link], [Link], [Link], [Link]), previously associated with kidney injury in animal models.^24^, ^25^ This demonstrates that soluble mediators released from the kidney recirculate and drive *de novo* expression of inflammatory genes within the organ, but that this can be alleviated by their removal from the perfusion circuit.

### 2.6 HA associated with a reduction in a delayed graft function–associated gene signature

**Figure 5:**
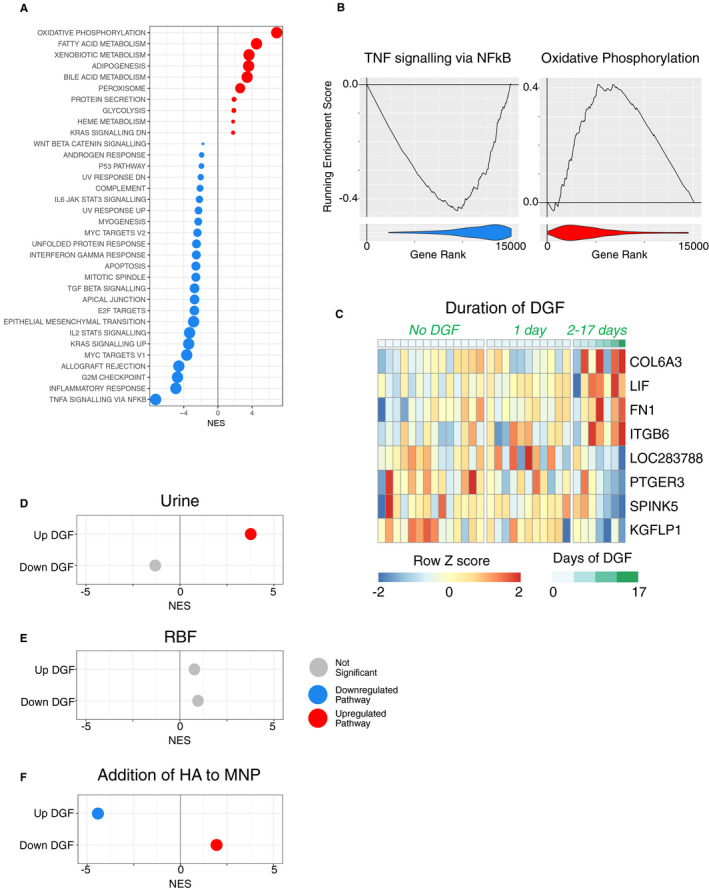
Addition of the hamadsorber reduces a DGF‐associated gene signature. A, GSEA analysis of the effect of the addition of HA to the transcriptome after 4 hours NMP against the hallmarks pathway of genesets. Only significant pathways are plotted. Red dots indicate positive enrichment and blue negative, the size of the dot is inversely correlated with the FDR q value and the position indicates the normalized enrichment score (NES). B, Enrichment plots from GSEA for key pathways from the Hallmark database for the comparison from A. The line indicates the running enrichment score and the violin plot indicates the distribution of the member genes of the geneset throughout the ranked gene list used in each analysis. C, Heatmap of the top four greatest positive (UP) and negative (DOWN) correlation with length of DGF and used as part of the gene expression signature for DGF. Full DGF signature given in supplemental data. D‐F, DGF‐associated gene signature was used with GSEA to investigate the expression of the signature in the correlation of 2‐hr urine output with 2‐hr transcriptome (D), correlation of 2‐hr RBF with 2‐hr transcriptome (E) and the effect of addition of HA on the transcriptome at 4 hours (F). Plots as for A

Gene set enrichment analysis showed a significant decrease in the “TNFα signaling via NFkB” pathway in NMP+HA kidneys compared with NMP alone (Figure 5A‐B). Notably, the presence of the HA not only reduced inflammatory gene expression within the kidney but also increased OXPHOS and fatty acid metabolism pathways, both of which contribute to energy generation (Figure 5A‐B). Therefore, the changes in gene expression pathways occurring with the HA would support the conclusion that its effects are clinically beneficial, since it reduced “TNFA signaling via NFkB” pathway genes and increased “OXPHOS” pathway genes, both of which were associated with a shorter duration of DGF (Figure 2B).

To further explore the relationship between transcriptional changes in NMP and clinical outcomes, we sought to curate a gene signature present in kidneys with DGF. We identified the top 100 positively (UP) and negatively (DOWN) regulated genes (ranked by log fold change) which correlated with the length of DGF in the 33 clinical trial samples (Figure 5C, Data [Link], [Link], [Link], [Link]). We found a significant enrichment of the gene signature associated with increased length of DGF in samples with a higher urine output (Figure 5D), suggesting that high urine output during NMP identifies kidneys at risk of more prolonged DGF. There was no statistically significant correlation between the DGF signature and the genes associated with higher renal blood flow (Figure 5E). We next assessed whether and how the addition of the HA to the NMP circuit affected the expression of DGF “UP” and “DOWN” gene signatures. Remarkably, this showed that the expression of the gene signature associated with increased length of DGF was significantly reduced by the addition of the HA, and the signature associated with decreased length of DGF was significantly increased by the addition of the HA (Figure 5F).

**Figure 6:**
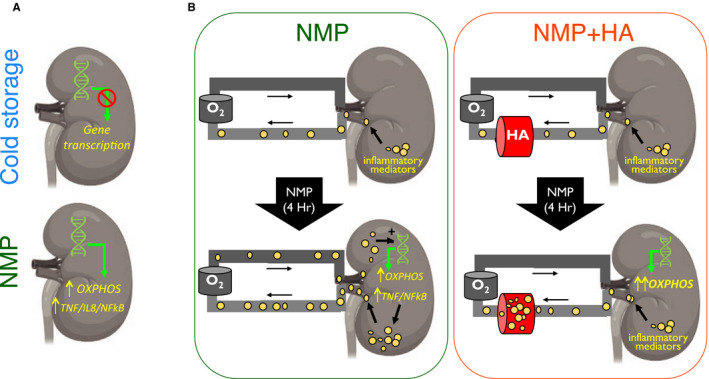
Graphical summary. A, During cold storage, there is a global reduction in transcription. During NMP the expression of genes in a number of pathways are upregulated including oxidative phosphorylation (OXPHOS) and inflammatory pathway genes such as *TNF*, *IL8*, and *NFkB*. B, left panel, During NMP, inflammatory mediators (yellow circles) are released from the kidney into the perfusion solution. They recirculate and stimulate pro‐inflammatory gene transcription in the kidney, and are associated with a reduction in energy pathway production genes, reducing ATP. Right panel, The presence of the hemoadorber (HA) breaks this inflammatory amplification loop

Overall, the transcriptional changes we have identified suggest that NMP has potential benefits over cold storage in terms of its effects on energy generation in the kidney. However, during perfusion, some bioactive molecules are released from the kidney into the perfusion circuit, generating an amplification loop that drives inflammation when they reenter the kidney. Removal of these molecules interrupts this loop, and may be useful to reduce inflammation and increase energy generation, further enhancing the beneficial effects of NMP and reducing susceptibility to DGF (Figure 6).

## 3 DISCUSSION

Our data show that cold storage is effective in limiting substantial changes in gene expression. This is in keeping with rodent models, where kidneys stored for up to 18 hours in cold storage showed little change in the expression of pro‐inflammatory cytokines, including IL1β, TNF, and IL6.^26^ Our use of unbiased, global transcriptional profiling rather than the measurement of a small number of candidate genes allowed us to analyze the expression of groups of genes found within specific pathways. This revealed a significant reduction in the expression of OXPHOS and glycolysis pathway genes in cold stored kidneys, potentially reducing the capacity of these kidneys to generate ATP, in line with a previous study showing reduced ATP in cold stored human kidneys.^27^ A number of pro‐inflammatory pathways were also downregulated during cold storage, including TNFα activation via NFkB and reactive oxygen species pathways which may be potentially beneficial. However, it may be that cold storage merely puts a temporary hold on these pathways, and that following reperfusion in the recipient, similar changes in inflammatory gene expression as observed in NMP would occur.

NMP had the opposite effect to cold storage on OXPHOS and glycolysis pathway genes, increasing their expression, with the potential to increase the cellular capacity to generate ATP and to restore homeostasis. Of note, hypothermic oxygenated perfusion of human kidneys has also been shown to increase ATP levels compared with cold storage ^27^, therefore the restoration of oxygenation may be the principle stimulus for these processes rather than normothermia. Nonetheless, these changes are likely to be beneficial to the organ given our observation of a negative correlation between increased expression of OXPHOS pathway genes and prolonged DGF.

A recent paper by Hameed *et al* ^28^ also performed transcriptomic analysis of NMP in three kidneys undergoing NMP. Unfortunately, the authors did not made their data publicly available, thus an in‐depth comparison with our dataset is not possible. However, their manuscript indicated an induction of immune response‐related genes during NMP, including IL1B, CXCL2, and TNF, which is concordant with our findings.

In our analysis of kidneys undergoing NMP in the context of a clinical trial, we correlated gene expression signatures with length of DGF rather than DGF incidence (defined as a need for dialysis within the first week posttransplant). Notably, kidneys with a duration of DGF of 1 day or less were highly transcriptionally similar to those with no DGF, likely reflecting dialysis requirement due to peri‐operative‐associated hyperkalemia rather than bona fide DGF. We found that a longer DGF was associated with higher expression of *TNFA signaling via NFkB* pathway genes and lower expression of genes associated with OXPHOS. These data suggest that kidneys with increased OXPHOS and decreased immune signaling may present better potential as donor kidneys, but this conclusion requires validation in a larger prospective study.

We also assessed the molecular processes that correlate with urine output and renal blood during NMP. These parameters have previously been used, along with a number of other measures, to generate a quality assessment score of perfused kidneys. High values of urine output and renal blood flow have been considered to reflect a more viable graft.^15^ Our data reveal that pathways correlating with high urine output and high renal blood flow differ, and in fact, these parameters demonstrate polar opposite associations with inflammatory pathways. High urine output was associated with higher expression of immune pathway genes while high renal blood flow was negatively correlated with these pathways. We also found that the DGF gene signature we generated was enriched in kidneys with a high urine output during NMP, suggesting that this parameter may identify kidneys at risk of a longer DGF, in contrast to the current dogma. One potential explanation is that a very high urine output reflects kidneys with more tubular damage that lack the capability to concentrate urine. Thus, there may be a “Goldilocks effect” with regard to urine output, where kidneys with low/little urine output are those with substantial abnormalities in the generation of filtrate, those with a very high urine output have substantial tubular damage precluding urine concentration. This hypothesis would need to be tested in a larger number of kidneys that were subsequently transplanted.

During 4 hours of NMP, there was an induction of inflammatory genes in the kidney. However, it is worth noting that current clinical practice involves 1 hour of NMP only, and this effect may not be evident over a shorter perfusion time. However, longer perfusion may have benefits in terms of restoration of oxygenation and energy generation, and in our experiment, the negative effects of immune gene induction could be substantially negated by the introduction of a cytosorb HA to the perfusion circuit. These data indicate that inflammatory mediators generated by the kidney during the course of NMP enter the perfusion circuit and are capable of exacerbating sterile inflammation, and that their removal ameliorates the induction of inflammation pathway genes observed during NMP. Importantly, we showed that the addition of the HA affects the expression of genes that are associated with worse outcomes clinically. Our DGF gene signature was derived from samples undergoing NMP as part of a clinical trial assessing its efficacy, allowing us to robustly link the changes we observed in our paired kidney studies with clinical endpoints in kidneys undergoing transplant. However, its application in the context of a clinical trial will be needed to definitively prove the utility of its application to kidneys pretransplant. The HA is nonspecific and may remove molecules which are helpful, in addition to those which are detrimental to the organ. We found that the net effect of the HA on the kidney transcriptome appeared beneficial, but it may be that a refinement to specifically remove proven, deleterious mediators may be even more effective.

The kidney pairs we used were from the same individual and were therefore genetically identical and had experienced a similar environment throughout the life of the donor. We confirmed that their time 0 transcriptome was extremely similar (Figure S2 A,B). However, these biopsies sample a small part of the kidney, and kidney pairs could be asymmetrically affected by pathology, for example, cysts, or small vessel disease. An additional caveat is that all kidneys used in the intervention experiments presented here were declined for transplantation and some represent transplanted organs at the lower end of the quality spectrum. Nevertheless, we observed highly reproducible results when comparing gene pathways in groups of five kidneys. We were also able to demonstrate that kidneys from DCD and DBD had a similar response to perfusion. By combining this with our curated post‐NMP DGF signature, we were able to predict potential clinical benefits. This experimental approach could be employed as a preclinical tool to screen future interventions for potential therapeutic efficacy to enable the rational selection of candidate interventions for clinical trials.

In summary, our study provides the first global transcriptional profile of human kidneys undergoing NMP, resolving the differing molecular pathways that are activated in NMP compared with cold storage, and showing that the deleterious effects of bioactive molecules produced or released from the kidney during NMP can be reversed by the addition of a HA. Furthermore, this intervention reduced the expression of genes associated with prolonged DGF providing a strong mechanistic rationale for applying such an intervention to a future clinical trial. Our data also have implications for perfusion strategies beyond the kidney, including in liver and lung transplantation, suggesting that the removal of bioactive molecules from perfusates should be investigated in these contexts where NMP is increasingly used. Finally, our study highlights the utility of global transcriptional profiling in paired kidneys for assessing novel interventions to perfused organs; transcriptional changes precede changes in protein abundance (traditionally used as biomarkers of kidney injury) and tens of thousands of gene transcripts can be readily measured. Thus, RNA measurement has the potential to provide an early, sensitive readout of cellular function of human organs retrieved for transplantation that can be applied to future studies.

## DISCLOSURE

The authors have no conflict of interest to disclose as described by the *American Journal of Transplantation*.

## AUTHOR CONTRIBUTIONS

J.R.F., S.H., M.L.N., and M.R.C. designed the study and interpreted the data. J.R.F., S.H., T.M., C.J.W, A.F., and T.C.D. performed the experiments. J.R.F., S.H., and M.R.C. created figures and tables. M.R.C. wrote the main manuscript. J.R.F. and S.H. created the methods and figure legends, J.R.F, S.H., and M.L.N. edited the manuscript.

## Data Availability

The transcriptional data for the experimental kidneys which supports the findings of this study are available at www.ncbi.nlm.nih.gov/geo under GSE121447. Remaining data will be made available upon request to the corresponding author.

## References

[1] Methven S , Steenkamp R , Fraser S . UK Renal Registry 19th Annual Report: Chapter 5 Survival and Causes of Death in UK Adult Patients on Renal Replacement Therapy in 2015: National and Centre‐specific Analyses. Nephron. 2017;137(1):117–150. 28930724 10.1159/000481367

[2] Summers DM , Johnson RJ , Allen J , et al. Analysis of factors that affect outcome after transplantation of kidneys donated after cardiac death in the UK: a cohort study. Lancet. 2010;376(9749):1303–1311. 20727576 10.1016/S0140-6736(10)60827-6

[3] Summers DM , Johnson RJ , Hudson A , Collett D , Watson CJ , Bradley JA . Effect of donor age and cold storage time on outcome in recipients of kidneys donated after circulatory death in the UK: a cohort study. Lancet. 2013;381(9868):727–734. 23261146 10.1016/S0140-6736(12)61685-7

[4] Friedewald JJ , Rabb H . Inflammatory cells in ischemic acute renal failure. Kidney Int. 2004;66(2):486–491. 15253694 10.1111/j.1523-1755.2004.761_3.x

[5] Kono H , Rock KL . How dying cells alert the immune system to danger. Nat Rev Immunol. 2008;8(4):279–289. 18340345 10.1038/nri2215 PMC2763408

[6] Berry M , Clatworthy MR . Immunotherapy for acute kidney injury. Immunotherapy. 2012;4(3):1–12. 22401637 10.2217/imt.11.175

[7] Parikh CR , Coca SG , Thiessen‐Philbrook H , et al. Postoperative biomarkers predict acute kidney injury and poor outcomes after adult cardiac surgery. J Am Soc Nephrol. 2011;22(9):1748–1757. 21836143 10.1681/ASN.2010121302 PMC3171945

[8] Hall IE , Yarlagadda SG , Coca SG , et al. IL‐18 and urinary NGAL predict dialysis and graft recovery after kidney transplantation. J Am Soc Nephrol. 2010;21(1):189–197. 19762491 10.1681/ASN.2009030264 PMC2799276

[9] Malyszko J , Lukaszyk E , Glowinska I , Durlik M . Biomarkers of delayed graft function as a form of acute kidney injury in kidney transplantation. Sci Rep. 2015;5:11684. 26175216 10.1038/srep11684 PMC4502393

[10] Hosgood SA , Nicholson ML . First in man renal transplantation after ex vivo normothermic perfusion. Transplantation. 2011;92(7):735–738. 21841540 10.1097/TP.0b013e31822d4e04

[11] Yong C , Hosgood SA , Nicholson ML . Ex‐vivo normothermic perfusion in renal transplantation: past, present and future. Curr Opin Organ Transplant. 2016;21(3):301–307. 27145197 10.1097/MOT.0000000000000316

[12] Fisher A , Andreasson A , Chrysos A , et al. An observational study of Donor Ex Vivo Lung Perfusion in UK lung transplantation: DEVELOP‐UK. Health Technol Assess. 2016;20(85):1–276. 10.3310/hta20850 PMC5136735 27897967

[13] Slama A , Schillab L , Barta M , et al. Standard donor lung procurement with normothermic ex vivo lung perfusion: A prospective randomized clinical trial. J Heart Lung Transplant. 2017;36(7):744–753. 28314503 10.1016/j.healun.2017.02.011

[14] Nasralla D , Coussios CC , Mergental H , et al. A randomized trial of normothermic preservation in liver transplantation. Nature. 2018;557(7703):50–56. 29670285 10.1038/s41586-018-0047-9

[15] Barlow AD , Hamed MO , Mallon DH , et al. Use of Ex Vivo Normothermic Perfusion for Quality Assessment of Discarded Human Donor Pancreases. Am J Transplant. 2015;15(9):2475–2482. 25989187 10.1111/ajt.13303 PMC7212093

[16] Hosgood SA , Saeb‐Parsy K , Hamed MO , Nicholson ML . Successful Transplantation of Human Kidneys Deemed Untransplantable but Resuscitated by Ex Vivo Normothermic Machine Perfusion. Am J Transplant. 2016;16(11):3282–3285. 27273794 10.1111/ajt.13906 PMC5096065

[17] Hosgood SA , Saeb‐Parsy K , Wilson C , Callaghan C , Collett D , Nicholson ML . Protocol of a randomised controlled, open‐label trial of ex vivo normothermic perfusion versus static cold storage in donation after circulatory death renal transplantation. BMJ Open. 2017;7(1):e012237. 10.1136/bmjopen-2016-012237 PMC5278243 28115329

[18] Hosgood SA , Moore T , Kleverlaan T , Adams T , Nicholson ML . Haemoadsorption reduces the inflammatory response and improves blood flow during ex vivo renal perfusion in an experimental model. J Transl Med. 2017;15(1):216. 29070045 10.1186/s12967-017-1314-5 PMC5657103

[19] Krebs HA . The history of the tricarboxylic acid cycle. Perspect Biol Med. 1970;14(1):154–170. 4923349 10.1353/pbm.1970.0001

[20] Hoogland ER , de Vries EE , Christiaans MH , Winkens B , Snoeijs MG , van Heurn LW . The value of machine perfusion biomarker concentration in DCD kidney transplantations. Transplantation. 2013;95(4):603–610. 23296150 10.1097/TP.0b013e31827908e6

[21] van Balkom BWM , Gremmels H , Ooms LSS , et al. Proteins in Preservation Fluid as Predictors of Delayed Graft Function in Kidneys from Donors after Circulatory Death. Clin J Am Soc Nephrol. 2017;12(5):817–824. 28476951 10.2215/CJN.10701016 PMC5477220

[22] Kogelmann K , Jarczak D , Scheller M , Druner M . Hemoadsorption by CytoSorb in septic patients: a case series. Crit Care. 2017;21(1):74. 28343448 10.1186/s13054-017-1662-9 PMC5366999

[23] David S , Thamm K , Schmidt BMW , Falk CS , Kielstein JT . Effect of extracorporeal cytokine removal on vascular barrier function in a septic shock patient. J Intensive Care. 2017;5:12. 28127437 10.1186/s40560-017-0208-1 PMC5251288

[24] Li L , Huang L , Vergis AL , et al. IL‐17 produced by neutrophils regulates IFN‐gamma‐mediated neutrophil migration in mouse kidney ischemia‐reperfusion injury. J Clin Invest. 2010;120(1):331–342. 20038794 10.1172/JCI38702 PMC2798679

[25] Hayama T , Matsuyama M , Funao K , et al. Benefical effect of neutrophil elastase inhibitor on renal warm ischemia‐reperfusion injury in the rat. Transplant Proc. 2006;38(7):2201–2202. 16980042 10.1016/j.transproceed.2006.06.094

[26] Saat TC , Susa D , Roest HP , et al. A comparison of inflammatory, cytoprotective and injury gene expression profiles in kidneys from brain death and cardiac death donors. Transplantation. 2014;98(1):15–21. 24901651 10.1097/TP.0000000000000136

[27] Ravaioli M , Baldassare M , Vasuri F , et al. Strategies to Restore Adenosine Triphosphate (ATP) Level After More than 20 Hours of Cold Ischemia Time in Human Marginal Kidney Grafts. Ann Transplant. 2018;23:34–44. 29326416 10.12659/AOT.905406 PMC6248038

[28] Hameed AM , Lu DB , Patrick E , et al. Brief Normothermic Machine Perfusion Rejuvenates Discarded Human Kidneys. Transplant Direct. 2019;5(11):e502. 31773055 10.1097/TXD.0000000000000944 PMC6831120

