# Supplementary material for: Cytokine absorption during human kidney perfusion reduces delayed graft function–associated inflammatory gene signature
Source: Am J Transplant. 2020 Nov 22;21(6):2188–99. doi: 10.1111/ajt.16371 (PMC8246774; doi:10.1111/ajt.16371)
Supplement: Supplementary file 3 [file AJT-21-2188-s002.docx]

**Supporting information**

**Methods**

**Study design**

**Research kidneys**

Ethical approval was granted from the national ethics committee in the UK REC reference (15/NE/0408). 10 pairs of human kidneys rejected for transplantation were recruited into the study. The study was divided into two parts. In the first study five pairs of kidney were randomly assigned to either cold storage or normothermic machine perfusion (NMP) for 2h. In the second study five pairs of kidneys were randomly assigned to either NMP with (NMP+HA) or without (NMP) the addition of a haemoadsorber (Cytosorb, Linc Medical, Leicester, UK) in the perfusion circuit (18). Kidneys were perfused for 4h.

**Kidney NMP transplant trial**

Ethical approval was grant by the East of England - Cambridge Central Research Ethics Committee (15/EE/0356) for a multicentre UK based randomised controlled, open label trial of the effect of NMP on initial graft function in donation after circulatory death (DCD) kidney transplantation. The study is open to eligible patients receiving a DCD kidney transplantation (Maastricht Categories III & IV). Patients are allocated at random in a 1:1 ratio to either static cold storage (CS) plus 1 hour of NMP (n = 200) or CS (n = 200) only. Wedge biopsies were collected post NMP and either snap frozen in liquid nitrogen or preserved in RNALater. Samples were then stored at -70°C. In this paper only a sub-sampling of the NMP arm was analysed. Trascriptomic data post-transplant was compared to the length of DGF which was defined as the number of days between transplantation and the last post-transplant dialysis session. Donor and recipient information is given in supplemental table 3. In this study samples were taken after 1 hour of NMP. Trial Registration Number: ISRCTN15821205

**Normothermic machine perfusion**

After a period of cold storage the kidneys were weighed and prepared for perfusion. The renal artery, vein and ureter were cannulated and kidneys flushed with 1L of cold Ringer’s solution to remove the preservation solution. Perfusion was carried out using an adapted paediatric cardiac bypass system (Medtronic, Bioconsole 560) as described previously (10). The perfusion system was primed with 300ml Ringer’s solution (Baxter Healthcare, Thetford UK), 15ml Mannitol 10% (Baxter Healthcare), 27 ml sodium bicarbonate 8.4% (Fresenius Kabi, Runcorn, UK), 3000iu heparin (LEO Pharma A/S, Ballerup, Denmark) and 6.6mg Dexamethasone (Hameln Pharmaceuticals, Hamelin, Germany). A unit of compatible packed red cells was then added. The red cell-based solution was oxygenated with a balance of 95% oxygen/5% CO_2_ at a flow rate of 0.1L/min and warmed to 35.5 - 36.5°C.

The red cell-based solution was circulated continually through the kidney via the renal artery at a mean arterial pressure of 85mmHg and pump speed of 1450RPM. A nutrient solution (Synthamin 17 10%, Baxter Healthcare, Thetford, UK) with 15ml of sodium bicarbonate 8.4% (B Braun, Melsungen , Germany) and 100IU of insulin added (Actrapid, Novo Nordisk, London, UK) was infused at a rate of 20ml/h, glucose 5% (Baxter Healthcare) at a rate of 5ml/h and Ringer’s solution was used to replace urine output (ml for ml). Epoprostenol sodium 0.5mg (Folan, Glaxo Wellcome UK Ltd, Uxbridge, UK) was infused at rate of 5ml/h throughout perfusion to enhance blood flow.

In the study with the addition of a HA a Cytosorb adsorber (Cytosorb 300ml Device, MedPass International Limited, Worrester, UK) was used. This was flushed and primed with Ringer’s solution then attached to the circuit by connecting it to the line from the oxygenator allowing the blood to flow through the adsorber back into the venous reservoir in parallel with the main flow to the renal artery

In the NMP vs CS the kidneys were perfused for 2 hrs with biopsies taken at 0 and 2hrs. In the NMP vs NMP + HA study biopsies were taken at 0, 2 and 4hrs.

**Normothermic Machine Perfusion Outcome Measures**

The renal blood flow (RBF) was recorded every 5min for the 30min and thereafter every 30min. Samples of perfusate were collected pre-perfusion and after each hour of perfusion for analysis of haematology and urea & electrolytes (U&Es). Urine samples were also collected hourly for the measurement of U&Es. Samples of perfusate were also retained for the measurement of cytokines. Perfusate was centrifuged at 1600rpm for 10min at 4°C. The supernatant was collected and frozen in liquid nitrogen then stored at –70°C until analysed.

Samples of arterial and venous perfusate were collected at 1 and 4h of perfusion for blood gas analysis (OPTI-CCS, Una Health, Stoke-on-Trent, UK). Oxygen consumption was calculated using the following equation;

Arterial content: C_a_O_2_ = (1.34 (Hb) x S_a_O2 x 0.01) + (0.023 x P_a_O_2_)

Venous content: C_v_O_2_ = (1.34 (Hb) x S_v_O2 x 0.01) + (0.023 x P_v_O_2_)

Oxygen consumption *= (*C_a_O_2_ x RBF) / (C_v_O_2_ x RBF)

Core biopsies were taken from each kidney at time 0 and after 2h of cold storage or perfusion in the first study and at time 0, 2h and 4h of perfusion in haemoadsorber study. Biopsies were stored in RNAlater solution (Invitrogen RNAlater™ Soln.) for fixing.

**RNA extraction**

RNA was extracted from biopsies stored in RNALater (Ambion) at -80C. Biopsies were removed from their storage solution and placed with 1ml Lysis Buffer (Ambion) in a MK28-R grinder tube (Bertin Instruments) and lysed using a Precellys 24 homogeniser (Bertin Instruments). Tubes were subsequently centrifuged at 1500xg for 4 minutes, the supernatant removed and the RNA extraction performed using a pure link RNA mini kit (Ambion) as per manufacturers instructions. Contaminating DNA was removed using TURBO DNase (Ambion) as per manufacturers instructions. Concentration of RNA was assessed using a Nanodrop Spectrophotometer (Thermo Scientific). Quality of RNA was assessed using a RNA nano Bioanalyzer kit (Agilent) using a Bioanalyzer 2100 (Agilent).

**RNA sequencing**

0.5µg of RNA was used for producing libraries for sequencing using TruSeq Stranded total RNA library prep kit (Illumina) as per manufactures instructions with a final PCR amplification of 14 cycles. Libraries were then sequenced on a Hiseq 4000 sequencer (Ilumina) by Genewiz. Further details are given the GEO series record. These data has been deposited under GSE121447.

**RNA sequencing analysis**

Following sequencing data was demultiplexed to give individual fastq files using Casava (Illumina). Fastq files were assessed for quality control purpose using FASTQC. The Fastq files were aligned to the human genome (Hg38) using Hisat2(31). All further analysis was carried out using the R statistical environment. A table of gene counts was produced using the featureCounts function within Rsubread and normalisation and differential gene expression analysis was carried out using DESeq2. For comparision between two groups (CS vs NMP, NMP vs NMP +HA) a factor based generalised liner model was fitted to the data and for comparison to a continuous output (Urine, RBF, length of DGF) a generalised liner model was fitted which took the variable of interest as continuous. For GSEA genes were ranked by the inverse of the p value with the sign of the log fold change and then ran against the hallmarks database within MSigDB using the GSEA program from the broad with the pre ranked option. ssGSEA was carried out using normalised counts from DESeq2. All GSEA outputs are given in supplementary file 2. All batch information, Fastq files, count tables and differential expression results are included in the GEO series record.

**String analysis**

The top 50 significant genes, ranked by log fold change for the effect of perfusion, were used to run STRING analysis. Gene names were converted into protein names and two proteins were considered connected if they had a mean interaction score of >0.7. Unconnected proteins were removed from the network and a force directed graph plotted. Network was subsequently clustered using k-means clustering and annotated using interpretation of GO terms associated with each cluster.

**Tissue protein extraction**

Total protein was extracted from tissue by homogenisation, using a precellys (Bertin instruments), of a small biopsy (approx. 27mm^3^) in T-PER tissue protein extraction reagent (thermo fisher scientific) with HALT protease inhibitor (thermo fisher scientific). Protein content was measured using by BCA analysis (thermo fisher scientific) and samples normalised.

**Cytokine measurements**

For all cytokine measurements analysis was carried out using DuoSet ELISA reagents (R&D) as per manufactures recommendations.

Supplemental Results

**Paired kidneys are genetically similar and a useful model for assessing interventions**

We reasoned that pairs of kidneys are genetically identical and experience a highly similar environment during the life of the donor, and this would be reflected in the transcriptome. To statistically test this assumption, we compared the transcriptome of all kidneys in the study by calculating the Euclidian distance between kidneys. This confirmed that the distance between a kidney pair was significantly shorter than that to all other kidneys in the study (**Figure S2A**). Next, we took the pairs of kidneys and randomly allocated them to one of two groups and performed differential gene expression analysis by fitting a generalised linear model which included a term for donor. Here we found that no individual genes were differentially expressed at baseline within a kidney pair (**Figure S2B**). Together, these data indicate that the inter-pair differences in gene expression are substantially less than intra-pair differences, and that kidney pairs are transcriptionally similar at baseline. Therefore, we could examine a pair of kidneys and apply a different intervention to each and contrast the outcome.

**Supplemental Figures and legends**

**Supplemental figure 1 – Schematic of all experiments carried out with time points for RNASeq indicted.**

**Supplemental figure 2 – Pairs of kidneys, regardless of donation type, are highly similar. A** – The Euclidian distance was calculated individually between each kidney and every other kidney in the dataset. The distance from each kidney to its contralateral pair (Pair) or the mean of the distance to all other kidneys (Non Pair) and the two points joined. A paired T test was used to test for difference between groups. **B** Each kidney within a pair was randomly assigned to either group A or B and differential expression analysis between the groups performed. Volcano plot shows changes in gene expression between the two groups. No genes passed a significance threshold of p < 0.05 post correction for multiple testing. **C** Same calculation as for B however separating groups by donation type instead. Distance to a kidneys contralateral pair was excluded for calculation of the mean. Difference between the groups tested using a paired T test. **D** Single sample GSEA was used for calculation of enrichment of each sample for the Hallmarked TNFa via NFkB pathway. This was ploted and the line links each kidney showing pre and post perfusion. The colour indicated donation type.

**Supplemental figure 3 - Correlation of transcriptome of kidneys prior to NMP with urine output after 2 hours. A –** Pathway enrichment for the effect of NMP on either 5 pairs of kidneys or 10 pairs of kidneys including the original 5. The normalised enrichment score for all the pathways which were significantly enriched in at least one comparison have been plotted and the spearman correlation calculated. The solid line is equal to y=x **B** Heatmap of all HSP genes ordered by urine output. **C** GSEA for the analysis of correlation of gene changes with urine output at 2 hours with the transcriptome either pre NMP (0hr) or after 2 hours (2hr) against the hallmarks database of gene sets. Only significant pathways are plotted. Red dots indicate positive enrichment and blue negative, the size of the dot is inversely correlated with the FDR q value and the position indicates the normalised enrichment score (NES). **D** Comparison of enrichment scores for correlation pre and post 2 hrs NMP with urine output by GSEA, against the Hallmarks database. Only pathways which are significant in at least 1 comparison have been plotted. Pathways which are altered between the conditions are labelled.

**Supplemental Figure 4 – Correlation of transcriptome prior to NMP with renal blood flow at 2 hrs. A** – Comparison of enrichment scores for correlation pre and post 2 hrs NMP with urine output by GSEA against the Hallmarks database. Only pathways which are significant in at least 1 comparison have been plotted. Pathways which are altered between the conditions are labelled. **B** - GSEA for correlation against the hallmarks database of genesets. Only significant pathways are plotted. Red dots indicate positive enrichment and blue negative, the size of the dot is inversely correlated with the FDR q value and the position indicates the normalised enrichment score (NES).

**Supplemental figure 5 – Effect of HA on cytokines and chemokines A** – Normalised gene expression profiles for all cytokines (Left panel) and chemokines (right panel) which were significantly altered by 4hrs post NMP. Selected cytokines annotated. Dashed line indicates LOESS regression line. Expression was normalised to respective time 0 for each group. Green line indicates NMP alone and orange with the addition of the haemoadsorber (HA). **B** - Heatmap showing Log2 fold change between pairs of kidneys relative to NMP alone for the indicated inflamazone related genes. **C** - Neutrophil recruiting chemokines plotted as for B.

**Supplementary Tables**

Supplementary Table 1 – Donor demographic in paired kidney NMP vs CS study

| Donor | 1 | 2 | 3 | 4 | 5 |
| --- | --- | --- | --- | --- | --- |
| Donor Type | DBD | DCD | DCD | DCD | DCD |
| Age | 75 | 76 | 51 | 62 | 70 |
| Gender | Male | Male | Female | Male | Female |
| CIT (Min) | 873 | 1481 | 1544 | 1402 | 602 |
| WIT (MIN) | 0 | 11 | 93 | 6 | 138 |
| Samples collected | Pre and 2hr | | | | |
| Reason offered for research | Poor function, poorly perfused, Histology, past medical history | | | | |

Supplementary Table 2 – Donor demographic in paired kidney NMP vs NMP + HA study

| Donor | 1 | 2 | 3 | 4 | 5 |
| --- | --- | --- | --- | --- | --- |
| Donor Type | DCD | DCD | DBD | DCD | DCD |
| Age | 66 | 62 | 67 | 61 | 70 |
| Gender | Male | Male | Male | Male | Male |
| CIT (Min) | 1375 | 1805 | 1791 | 1659 | 606 |
| WIT (MIN) | 12 | 41 | 0 | 16 | 11 |
| Samples collected | Pre, 2hr, 4hr | | | | |
| Reason offered for research | Poorly perfused, poorly flushed, long CIT, long WIT | | | | |

Supplementary Table 3 – Donor and recipient information for samples taken from NMP arm of randomised clinical trial comparing NMP with cold storage. Samples used for the analysis are a sub sample of the trial.

|  | Donor information |
| --- | --- |
| Donor Age | Mean=54.4, SD=13.6 |
| Donor Gender | Male=20, Female=12 |
| Donor Type | All DCD |
| Recipient Age | Mean=56.2, SD=12.5 |
| CIT (min) | Mean=686.6, SD=204.69 |
| WIT (min) | Mean = 11.6, SD = 2.8 |
| DGF duration (days) | No DGF=14  1 day=10  2 days=1  3 days =2  > 3 days = 5 |

Supplemental Table 4 – Urine analysis for 4 hr time point of NMP vs NMP + HA study

|  | NMP | NMP+HA |
| --- | --- | --- |
|  | **4h** | **4h** |
| Creatinine clearance 4h (ml/min/100g) | 0.546 ± 0.23 | 0.640 ± 0.56 |
| Fractional Excretion of Na+ (%) 4h | 54.925 ± 8.775 | 75.35 ± 4.35 |

Supplementary Table 5 – Levels of pH, bicarbonate, base excess and oxygen consumption during normothermic machine perfusion (NMP)

|  | NMP | | | NMP+HA | | |
| --- | --- | --- | --- | --- | --- | --- |
|  | **Pre** | **1h** | **4h** | **Pre** | **1h** | **4h** |
| pH | 7.29 ± 0.1 | 7.40 ± 0.1 | 7.47 ± 0.1 | 7.26 ± 0.1 | 7.36 ± 0.1 | 7.43 ± 0.1 |
| Bicarbonate  (mmol/L) | 38.7 ± 12.5 | 28.5 ± 5.7 | 21.0 ± 3.6 | 31.6 ± 7.4 | 26.0 ± 6.2 | 24.2 ± 8.2 |
| Base excess  (mmol/L) | 9.8 ± 12.3 | 3.3 ± 6.8 | -1.9 ± 4.0 | 3.0 ± 7.1 | 0.2 ± 6.2 | -0.55 ± 10.0 |
| Oxygen consumption  (ml/min/100g) |  | 1.19 ± 0.10 | 1.24 ± 0.16 |  | 1.41 ± 0.24 | 1.27 ± 0.16 |

**Supplemental Files**

Supplemental file 1 – excel file of the gene lists used for DGF.

Supplemental file 2 – excel file of all the GSEA outputs for each figure.
